# Apical Sparing e Hiperrefringência Miocárdica: Nem Tudo que Reluz é Amiloidose Cardíaca

**DOI:** 10.36660/abc.20250198

**Published:** 2025-12-08

**Authors:** Ana Paula Morais Barros, Cristhian Espinoza Romero, Juliano Novaes Cardoso, Fábio Fernandes, Viviane Tiemi Hotta

**Affiliations:** 1 Hospital das Clínicas Faculdade de Medicina Universidade de São Paulo São Paulo SP Brasil Instituto do Coração do Hospital das Clínicas da Faculdade de Medicina da Universidade de São Paulo, São Paulo, SP – Brasil; 2 Faculdade Santa Marcelina São Paulo SP Brasil Faculdade Santa Marcelina, São Paulo, SP – Brasil

**Keywords:** Deformação Longitudinal Global, Amiloidose, Ecocardiografia Doppler

## Introdução

Com os recentes avanços no diagnóstico e tratamento da amiloidose cardíaca (AC), tem sido amplamente discutido o papel dos métodos complementares em imagem cardíaca para o reconhecimento precoce dessa doença. Nesse contexto, a hiperrefringência miocárdica e a preservação da contratilidade do ápice do ventrículo esquerdo ( *apical sparing* ; AS), evidenciadas pelo ecocardiograma transtorácico (ETT) com análise do *strain* miocárdico (STE), se configuram como achados que frequentemente levantam a suspeita de AC.

Entretanto, apesar dessa associação, a heterogeneidade desse achado na AC, dependendo da população estudada, além da sua apresentação em outras condições, leva ao questionamento a respeito da real acurácia do AS para o diagnóstico dessa doença. Através do relato de três casos clínicos de pacientes portadores de condições clínicas diversas - cardiomiopatia hipertrófica (CMH), doença renal crônica (DRC) e uso de esteroides anabolizantes - em que foram descritos a hiperrefringência miocárdica e o AS à ecocardiografia, é proposta uma breve discussão a respeito da fisiopatologia desses achados e de suas limitações para o diagnóstico de AC.

## Caso 1

Paciente de 28 anos, sexo masculino, portador de CMH forma não obstrutiva diagnosticada aos 15 anos de idade. Foi realizado teste genético que evidenciou variante do gene *MYBPC3* em heterozigose, sendo classificada como variante patogênica com associação definitiva com CMH.^1 , 2^

**Figura 1:**
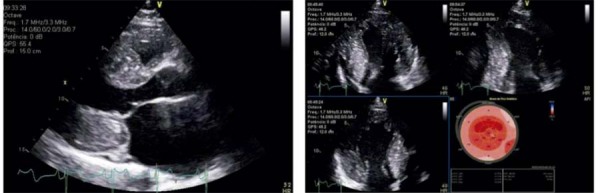
– Imagens ecocardiográficas de paciente com CMH sarcomérica. Aumento importante da espessura miocárdica das paredes septal anterior e lateral posterior, com aspecto brilhante e hiperrefringente, além de aumento do átrio esquerdo ao corte paraesternal longitudinal (A) e aos cortes apicais (B), além de padrão de apical sparing com deformação longitudinal preservada em segmentos médios e apicais (B). CMH: cardiomiopatia hipertrófica; AE: átrio esquerdo; VE: ventrículo esquerdo; VD: ventrículo direito.

ETT realizado em 2023 evidenciou dilatação importante do átrio esquerdo (volume = 89 ml/m^2^), VE com diâmetros diastólico e sistólico finais de 45 mm por 29 mm, respectivamente, espessura do septo interventricular (SIV) de 20 mm e espessura de parede posterior (PP) de 24 mm e aspecto hiperrefringente do músculo cardíaco caracterizado por infiltração granular das paredes ventriculares ( *granular sparkling* ) ( Figura 1 ). A função sistólica estava preservada, não sendo observadas alterações da mobilidade segmentar das paredes miocárdicas. O gradiente sistólico máximo em via de saída do VE foi estimado em 14 mmHg, em repouso e em 27 mmHg após manobra de Valsalva. A análise da função diastólica foi compatível com padrão restritivo (disfunção diastólica importante).

**Figura 2:**
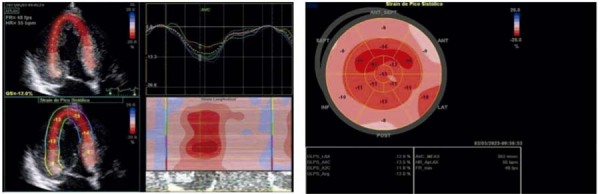
– Imagens ecocardiográficas da análise da mecânica cardíaca pela técnica de speckle tracking de paciente com CMH sarcomérica. Observa-se redução do strain segmentar em segmentos basais dos cortes apicais evidenciado pelas cores mais claras (A) e imagem paramétrica com padrão de apical sparing, evidenciado por deformação longitudinal preservada em segmentos médios e apicais (B). CMH: cardiomiopatia hipertrófica.

O *Strain* Global Longitudinal (SGL) absoluto do ventrículo esquerdo avaliado pela técnica *Speckle Tracking* foi estimado em 12%. Foi observada a redução marcada do *strain* nos segmentos basais e relativa preservação dos segmentos apicais (AS), com índice de AS relativo de 1.27 ( Figura 2 ).

## Caso 2

**Figura 3:**
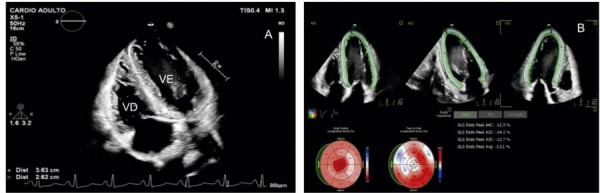
– Imagens ecocardiográficas da análise da mecânica cardíaca pela técnica de speckle tracking de paciente portador de insuficiência mitral moderada e DRC. Observa-se aumento da espessura miocárdica, com aspecto hiper-refringente. O strain longitudinal global foi calculado em 13,1%, apesar da fração de ejeção preservada (A). Imagem paramétrica com padrão de apical sparing, evidenciado por deformação longitudinal preservada em segmentos apicais (B). DRC: doença renal crônica; VE: ventrículo esquerdo; VD: ventrículo direito.

Paciente de 34 anos, sexo masculino, hipertenso e portador de DRC dialítica há 6 anos, foi encaminhado ao serviço de cardiologia por insuficiência mitral moderada evidenciada em ETT solicitado no contexto de pré-operatório de transplante renal, sendo descartadas outras cardiopatias. ETT realizado em 2024 evidenciou aumento de átrio esquerdo (volume = 55 ml/m^2^), VE com diâmetros sistólico e diastólico finais de 48 mm por 34 mm, respectivamente, espessura do SIV e espessura de PP de 12 mm, espessamento discreto valvar mitral insuficiência de grau moderado ao Doppler com *Vena contracta* estimada em 4 mm. Análise pelo STE demonstrou SGL = 13,1%, com o achado de AS e redução da deformação longitudinal segmentos basais e médios, com índice de *as* relativo de 1.04 ( Figura 3 ).

## Caso 3

Paciente de 49 anos, sexo masculino, atleta maratonista há 20 anos, assintomático do ponto de vista cardiovascular e sem comorbidades conhecidas. Relatava uso de esteroides anabolizantes (EA) com a finalidade de aumento de desempenho físico em atividades esportivas. Encaminhado para atendimento ambulatorial junto à cardiologia para rastreio de possível cardiotoxicidade relacionada ao uso de EA, tendo sido descartadas outras causas de cardiopatia.

**Figura 4:**
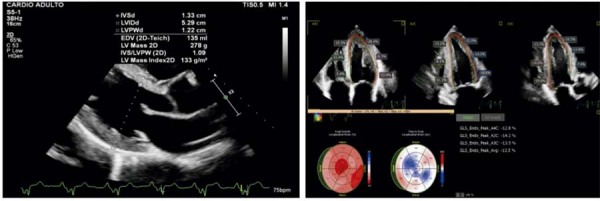
– Imagens ecocardiográficas da análise da mecânica cardíaca pela técnica de speckle tracking de paciente usuário de esteroides anabolizantes. Hipertrofia concêntrica do ventrículo esquerdo ao corte paraesternal longitudinal (A) e imagem paramétrica com padrão de apical sparing, evidenciado por deformação longitudinal preservada em segmentos apicais (B). VE: ventrículo esquerdo.

O ETT evidenciou hipocontratilidade difusa do VE, com comprometimento discreto de sua função sistólica (FEVE:0,49). O AE apresentou volume de 33 ml/m^2^ e foi observada hipertrofia concêntrica, com SIV de 13 mm e PP de 12 mm. A análise da mecânica cardíaca demonstrou redução do *strain* , com AS ( Figura 4 ).

## Discussão

A análise do *strain* miocárdico pela técnica de STE utiliza algoritmos de processamento de imagem obtidos através do ecocardiograma bidimensional. Esse método fornece informações sobre a deformação miocárdica global e segmentar, por meio do rastreamento quadro a quadro de marcadores acústicos ao longo do ciclo cardíaco, sendo capaz de detectar alterações iniciais da função ventricular, mesmo antes das demonstradas pelo ecocardiograma bidimensional convencional.^3^ O valor normal do SGL do ventrículo esquerdo varia entre 15,9% e 22,1%.^4^

Além da detecção de alterações precoces da função ventricular, esse método tem sido utilizado em diversas áreas da cardiologia, como na investigação diagnóstica do aumento da espessura miocárdica, na cardio-oncologia, estenose aórtica e cardiopatia hipertensiva. O STE vem ganhando notoriedade através da identificação da disfunção sistólica no contexto de fração de ejeção preservada, auxiliando no diagnóstico diferencial do aumento de espessura miocárdica ventricular.

Além do valor prognóstico, diferenças no padrão de distribuição do SGL, têm sido utilizadas como método útil no auxílio diagnóstico entre as diferentes etiologias de miocardiopatias. Enquanto a ressonância magnética cardíaca (RMC) pode sugerir a suspeita de AC e a cintilografia com pirofosfato pode definir o diagnóstico de amiloidose por transtirretina após a exclusão de amiloidose por cadeias leves, o ETT permanece como o exame inicialmente realizado e que levanta a suspeita dessa condição.

O padrão de *strain* denominado como AS tem sido classicamente relacionado com achado característico da AC. Nesse padrão, a razão entre a média dos valores de strain (itálico) longitudinal dos segmentos apicas e a médios dos valores de strain (itálico) longitudinal basais e médios acima de 1, foi descrito como capaz de diferenciar a AC de outros fenótipos de hipertrofia do VE com boa sensibilidade e especificidade.^5^ Uma hipótese proposta para esse fenômeno sugere a existência de um gradiente na infiltração amiloide, com menor envolvimento do ápice, que pode ser devido a diferenças regionais na perfusão ou composição tecidual do miocárdio.^6^

Entretanto, apesar da redução do SGL com padrão de AS ser tipicamente associado à AC, achado semelhante também pode ser encontrado em diferentes cenários, como doença renal em estágio avançado, estenose aórtica importante, além de outras miocardiopatias, como a própria CMH e na cardiotoxicidade induzida pelo uso de EA.

Em 2024, foi publicado um estudo multicêntrico, internacional, realizado com o intuito de avaliar a acurácia diagnóstica do AS na AC. Foram avaliados 544 ecocardiogramas de pacientes com AC confirmada, 200 ecocardiogramas de pacientes com elevada suspeita clínica e/ou ecocardiográfica, porém com diagnóstico descartado de AC e 174 ecocardiogramas de indivíduos saudáveis. Um dos parâmetros avaliados foi a relação *apical sparing ratio* (ASR) - razão da média do *strain* longitudinal dos segmentos apicais pela média do *strain* longitudinal dos segmentos basais e médios. Utilizando o ponto de corte de 1.67 no ASR, a sensibilidade encontrada foi de apenas 72%, enquanto a especificidade foi de 66%. Utilizando o valor de ASR ≥2.0, a acurácia diagnóstica permaneceu baixa, com o padrão de AS sendo identificado em 32% dos pacientes do grupo controle e em 6% dos pacientes do grupo controle saudável.^7^

Tanto no caso do paciente portador de CMH sarcomérica quanto no do paciente com DRC, os padrões de AS identificados apresentaram valores de ASR de 1,27 e 1,04, respectivamente. Essas relações, abaixo do ponto de corte de 1,67 sugerido no estudo mencionado — o qual visa conferir maior especificidade desse achado ao diagnóstico de AC — sugerem que talvez seja necessária a adoção de outros parâmetros que auxiliem na interpretação do AS no diagnóstico diferencial das cardiopatias.

Outro estudo avaliando 547 ecocardiogramas demonstrou que, apesar de o achado de AS aumentar a probabilidade de diagnóstico de amiloidose, apresentou sensibilidade e especificidade modestas. O mesmo estudo avaliou que a utilização da razão entre a fração de ejeção ventricular esquerda e o SGL poderia ter uma maior acurácia do que o AS no diagnóstico da AC.^8^

Além do AS, o aspecto granular do miocárdio ( *granular sparkling)* também é descrito como outro achado característico da AC na avaliação ecocardiográfica. Essa descrição da textura miocárdica na amiloidose decorre do aumento da ecogenicidade em virtude da deposição das fibrilas amiloides no músculo cardíaco.^9^ Entretanto, o *granular sparkling* também não pode ser considerado um achado específico da amiloidose, podendo ser descrito em outras condições, como na miocardite com fibrose importante, em outras doenças infiltrativas do miocárdio, CMH,^10^ DRC e cardiotoxicidade induzida pelo uso de EA, o que pode ser ilustrado através do casos apresentados.

Em uma publicação que analisou a presença de dano miocárdico em atletas usuários de esteroides anabolizantes (EA) na fase *off-cycle* , se observou uma redução desproporcional do SGL nessa população, em comparação com atletas não usuários. Nesse estudo, a maioria dos indivíduos apresentava preservação do *strain* na região apical do ventrículo, caracterizando o padrão de AS.^1 1^ Em concordância com esse achado, o paciente descrito no Caso 3 apresentou redução do *strain* predominantemente nas porções média e basal do VE, com preservação da contratilidade apical.

## Conclusão

Como nos casos relatados acima, o achado do AS foi descrito em pacientes portadores de CMH sarcomérica confirmada, DRC avançada e cardiotoxicidade relacionada ao uso de EA. Diante do exposto, apesar da clássica associação entre granular *sparkling* e AS à AC, é possível observar que tais achados talvez apresentem limitações significativas. Assim como nos casos descritos, torna-se evidente a necessidade de integrar parâmetros clínicos, genéticos, familiares e análise de outros exames de imagem para uma avaliação mais acurada quanto ao diagnóstico diferencial de determinada miocardiopatia frente a esses achados ecocardiográficos.
