# Supplementary material for: Apical Sparing e Hiperrefringência Miocárdica: Nem Tudo que Reluz é Amiloidose Cardíaca
Source: Arq Bras Cardiol. 2025 Dec 8;122(11):e20250198. [Article in Portuguese] doi: 10.36660/abc.20250198 (PMC12711223; doi:10.36660/abc.20250198)
Supplement: Translation

# Translation: Apical Sparing and Granular Sparkling: Not Everything that Glitters Is Cardiac Amyloidosis

**DOI:** 10.36660/abc.20250198i

**Authors:** Ana Paula Morais Barros, Cristhian Espinoza Romero, Juliano Novaes Cardoso, Fábio Fernandes, Viviane Tiemi Hotta

**Affiliations:** 1 Brazil Instituto do Coração do Hospital das Clínicas da Faculdade de Medicina da Universidade de São Paulo, São Paulo, SP – Brazil; 2 Brazil Faculdade Santa Marcelina, São Paulo, SP – Brazil

**Keywords:** Global Longitudinal Strain, Amyloidosis, Doppler Echocardiography

## Introduction

With recent advances in the diagnosis and treatment of cardiac amyloidosis (CA), the role of complementary cardiac imaging methods for the early recognition of this disease has been widely discussed. In this context, myocardial hyperrefringence and preservation of left ventricular apex contractility (apical sparing; AS), evidenced by transthoracic echocardiography (TTE) with myocardial strain analysis (STE), are findings that frequently raise suspicion of CA.

However, despite this association, the heterogeneity of this finding in CA, depending on the population studied, in addition to its presentation in other conditions, leads to questions regarding the real accuracy of AS for the diagnosis of this disease. Through the report of three clinical cases of patients with different clinical conditions - hypertrophic cardiomyopathy (HCM), chronic kidney disease (CKD), and use of anabolic steroids - in which myocardial hyperrefringence and AS were described on echocardiography, a brief discussion is proposed regarding the pathophysiology of these findings and their limitations for the diagnosis of CA.

## Case 1

A 28-year-old male patient with non-obstructive HCM was diagnosed at the age of 15. Genetic testing revealed a heterozygous variant of the *MYBPC3* gene, which was classified as a pathogenic variant with a definitive association with HCM.^1 , 2^

TTE performed in 2023 showed significant left atrial dilation (volume = 89 ml/m^2^), LV with end-diastolic and end-systolic diameters of 45 mm by 29 mm, respectively, interventricular septal (IVS) thickness of 20 mm and posterior wall (PP) thickness of 24 mm, and hyperrefringent appearance of the cardiac muscle characterized by granular infiltration of the ventricular walls (granular sparkling) ( Figure 1 ). Systolic function was preserved, with no changes in segmental myocardial wall mobility observed. The maximum systolic gradient in the LV outflow tract was estimated at 14 mmHg at rest and 27 mmHg after the Valsalva maneuver. Diastolic function analysis was consistent with a restrictive pattern (significant diastolic dysfunction).

**Figure 1:** – Echocardiographic images of a patient with sarcomeric HCM. Significant increase in myocardial thickness of the anterior and posterior lateral septal walls, with a shiny and hyperrefringent appearance, in addition to enlargement of the left atrium in the parasternal longitudinal view (A) and apical views (B), in addition to an apical sparing pattern with preserved longitudinal deformation in the middle and apical segments (B). HCM: hypertrophic cardiomyopathy; LA: left atrium; LV: left ventricle; RV: right ventricle.

The absolute Global Longitudinal Strain (GLS) of the left ventricle assessed by the Speckle Tracking technique was estimated at 12%. A marked reduction in strain was observed in the basal segments and relative preservation of the apical segments (AS), with a relative AS index of 1.27 ( Figure 2 ).

**Figure 2:** – Echocardiographic images of cardiac mechanics analysis using speckle tracking in a patient with sarcomeric HCM. Reduced segmental strain is observed in the basal segments of the apical sections, evidenced by lighter colors (A), and parametric images with an apical sparing pattern, evidenced by preserved longitudinal deformation in the middle and apical segments (B). HCM: hypertrophic cardiomyopathy.

## Case 2

A 34-year-old male patient, hypertensive and with CKD on dialysis for 6 years, was referred to the cardiology department due to moderate mitral regurgitation evidenced on TTE requested in the context of preoperative kidney transplantation, with other heart diseases being ruled out. TTE performed in 2024 showed left atrial enlargement (volume = 55 ml/m^2^), LV with end-systolic and end-diastolic diameters of 48 mm by 34 mm, respectively, IVS thickness and PP thickness of 12 mm, discrete mitral valve thickening and moderate mitral valve regurgitation on Doppler with *Vena contracta* estimated at 4 mm. STE analysis demonstrated SGL = 13.1%, with the finding of AS and reduced longitudinal deformation in the mid and basal portions, with a relative apical sparing index of 1.04 ( Figure 3 ).

**Figure 3:** – Echocardiographic images of cardiac mechanics analysis using speckle tracking in a patient with moderate mitral regurgitation and CKD. Increased myocardial thickness with a hyperrefringent appearance is observed. Global longitudinal strain was calculated at 13.1%, despite preserved ejection fraction (A). Parametric image with an apical sparing pattern, evidenced by preserved longitudinal deformation in apical segments (B). CKD: chronic kidney disease; VE: left ventricle; VD: right ventricle.

## Case 3

A 49-year-old male patient, a marathon runner for 20 years, was asymptomatic from a cardiovascular perspective and had no known comorbidities. He reported using anabolic steroids (AS) to increase physical performance in sports activities. He was referred to outpatient cardiology for screening for possible cardiotoxicity related to AS use, and other causes of heart disease were ruled out.

TTE revealed diffuse LV hypocontractility, with mild impairment of systolic function (LVEF: 0.49). The LA had a volume of 33 ml/m^2^, and concentric hypertrophy was observed, with an IVS of 13 mm and a PP of 12 mm. Cardiac mechanics analysis demonstrated reduced strain, with AS ( Figure 4 ).

**Figure 4:** – Echocardiographic images of cardiac mechanics analysis using speckle tracking in a patient using anabolic steroids. Concentric left ventricular hypertrophy in the longitudinal parasternal view (A) and parametric image with an apical sparing pattern, evidenced by preserved longitudinal deformation in apical segments (B). LV: left ventricle.

## Discussion

Strain is a method that uses image processing algorithms obtained through two-dimensional echocardiography. This method provides information on global and segmental myocardial deformation through frame-by-frame tracking of acoustic markers throughout the cardiac cycle, capable of detecting early changes in ventricular function, even before those demonstrated by conventional two-dimensional echocardiography.^3^The normal value for left ventricular GLS ranges from 15.9% to 22.1%.^4^

In addition to detecting early changes in ventricular function, this method has been used in several areas of cardiology, such as the diagnostic investigation of increased myocardial thickness, cardio-oncology, aortic stenosis, and hypertensive heart disease. STE has been gaining notoriety for identifying systolic dysfunction in the context of preserved ejection fraction, aiding in the differential diagnosis of increased ventricular myocardial thickness.

In addition to its prognostic value, differences in the distribution pattern of GLS have been used as a useful diagnostic tool for different etiologies of cardiomyopathies. While cardiac magnetic resonance imaging (CMR) may suggest CA, and pyrophosphate scintigraphy may confirm the diagnosis of transthyretin amyloidosis after ruling out light chain amyloidosis, TTE remains the initial test that raises suspicion of this condition. The strain pattern known as SA has been classically associated with a characteristic finding of CA. In this pattern, a ratio of mean apical longitudinal systolic strain to basal and mean longitudinal systolic strain greater than 1 has been described as capable of differentiating CA from other LV hypertrophy phenotypes with good sensitivity and specificity.^5^One hypothesis proposed for this phenomenon suggests a gradient in amyloid infiltration, with less involvement of the apex, which may be due to regional differences in myocardial perfusion or tissue composition.^6^

However, although reduced GLS with an AS pattern is typically associated with CA, a similar finding can also be found in different scenarios, such as advanced kidney disease, significant aortic stenosis, and other cardiomyopathies, such as HCM itself, and AS-induced cardiotoxicity.

In 2024, a multicenter, international study was published to evaluate the diagnostic accuracy of AS in CA. A total of 544 echocardiograms from patients with confirmed CA, 200 echocardiograms from patients with high clinical and/or echocardiographic suspicion but with a ruled-out diagnosis of CA, and 174 echocardiograms from healthy individuals were evaluated. One of the parameters evaluated was the apical sparing ratio (ASR)—the ratio of the mean longitudinal strain of the apical segments to the mean longitudinal strain of the basal and mid-segments. Using the ASR cutoff of 1.67, sensitivity was only 72%, while specificity was 66%. Using an ASR value ≥2.0, diagnostic accuracy remained low, with the AS pattern identified in 32% of patients in the control group and in 6% of patients in the healthy control group.^7^

In both the patient with sarcomeric HCM and the patient with CKD, the apical sparing patterns identified presented ASR values of 1.27 and 1.04, respectively. These ratios, below the 1.67 cutoff suggested in the aforementioned study—which aims to provide greater specificity for this finding in the diagnosis of CA—suggest that it may be necessary to adopt other parameters to aid in the interpretation of AS in the differential diagnosis of heart disease.

Another study evaluating 547 echocardiograms demonstrated that, although the finding of AS increases the likelihood of diagnosing amyloidosis, it had modest sensitivity and specificity. The same study assessed that using the ratio between ventricular function and GLS could be more accurate than AS in diagnosing CA.^8^

In addition to AS, the granular appearance of the myocardium (granular sparkling) is also described as another characteristic finding of CA on echocardiographic evaluation. This description of myocardial texture in amyloidosis results from increased echogenicity due to the deposition of amyloid fibrils in the cardiac muscle.^9^However, granular sparkling cannot be considered a finding specific to amyloidosis, and can be described in other conditions, such as myocarditis with significant fibrosis, other infiltrative myocardial diseases, HCM,^10^CKD, and cardiotoxicity induced by the use of AS, which can be illustrated by the cases presented.

In a publication that analyzed the presence of myocardial damage in athletes using anabolic steroids (AS) in the off-cycle phase, a disproportionate reduction in GLS was observed in this population, compared to athletes who did not use them. In this study, most individuals had preserved strain in the apical region of the ventricle, characterizing the apical sparing pattern.^1 1^In agreement with this finding, the patient described in Case 3 presented reduced strain predominantly in the mid and basal portions of the LV, with preserved apical contractility.

## Conclusion

As in the cases reported above, the finding of AS has been described in patients with confirmed sarcomeric HCM, advanced CKD, and cardiotoxicity related to AS use. Given the above, despite the classic association between granular sparkling and AS in CA, it is possible to observe that such findings may have significant limitations. As in the cases described, the need to integrate clinical, genetic, and family parameters and analysis of other imaging exams becomes evident for a more accurate assessment of the differential diagnosis of a given cardiomyopathy in light of these echocardiographic findings.
